# Impact of the COVID-19 Pandemic on the Reported Incidence of Select Bacterial Enteric Diseases in Canada, 2020

**DOI:** 10.1089/fpd.2022.0064

**Published:** 2023-03-09

**Authors:** Brendan Dougherty, Russell O. Forrest, Courtney R. Smith, Vanessa Morton, Lauren M. Sherk, Brent Avery, Ashley Kearney, Sara Christianson, Celine Nadon, M. Kate Thomas

**Affiliations:** ^1^ Public Health Agency of Canada, Centre for Food-Borne, Environmental & Zoonotic Infectious Diseases, Food-Borne Disease and Antimicrobial Resistance Surveillance Division, Guelph, Canada.; ^2^ Public Health Agency of Canada, Centre for Food-Borne, Environmental & Zoonotic Infectious Diseases, Outbreak Management Division, Ottawa, Canada.; ^3^ Public Health Agency of Canada, National Microbiology Laboratory, Division of Enteric Diseases, Winnipeg, Canada.

**Keywords:** COVID-19, foodborne illness, trends

## Abstract

The aim of this study was to describe the impact of the COVID-19 pandemic on reported cases and clusters of select enteric diseases in Canada, for the period of March 2020 to December 2020. Weekly counts of laboratory confirmed cases of *Salmonella*, *Shigella*, Shiga toxin–producing *Escherichia coli* (STEC), and *Listeria monocytogenes* were obtained from laboratory surveillance data. These data were supplemented with epidemiological information on the suspected source of illness, collected for cases identified within whole genome sequencing clusters. Incidence rate ratios were calculated for each pathogen. All data were compared with a prepandemic reference period. Decreases in the number of reported cases in 2020 compared with the previous 5-year period were noted for *Salmonella*, *Shigella*, *Escherichia coli* O157, and non-O157 STEC. Reported number of cases for *L. monocytogenes* in 2020 remained similar to those of the previous 5-year period. There was a considerable decline (59.9%) in the number of cases associated with international travel compared with a 10% decline in the number of domestic cases. Comparison of reported incidence rates of clustered versus sporadic cases for each pathogen showed little variation. This study represents the first formal assessment of the impact of COVID-19 on reported enteric diseases in Canada. Reported case counts across several pathogens saw notable declines in 2020 compared with prepandemic levels, with restrictions on international travel playing a key role. Additional research is needed to understand how limitations on social gatherings, lock downs, and other public health measures have impacted enteric diseases.

## Introduction

The COVID-19 pandemic has resulted in varied and widespread impacts on the Canadian population. In March 2020, when the pandemic was first declared by the World Health Organization (Cucinotta and Vanelli, 2020), federal, provincial and territorial, and local governments began implementing measures to reduce the spread of the SARS-CoV-2 virus. These measures included closures of schools and nonessential business, and encouragement of remote working. In-person social interactions were actively discouraged, and limits to the size of gatherings were implemented and imposed to varying degrees.

In addition, unprecedented travel restrictions were put into place. The number of international flights both into and out of Canada was limited, and the land border with the United States was closed for the first time in Canadian history (Detsky and Bogoch, 2020).

Evidence globally has suggested that the COVID-19 pandemic, and its associated public health measures, has had a significant impact on enteric diseases. Decreases in shigellosis have been noted in Israel (Bassal et al., 2021), reductions in campylobacteriosis and salmonellosis have been reported in Switzerland (Steffen et al., 2020), and significant declines in the number of gastrointestinal outbreaks were noted in England (Love et al., 2022). Similar decreases in enteric disease activity have been reported in the United States (Ray et al., 2021), China (Wang et al., 2021), Spain (de Miguel Buckley et al., 2020), Taiwan (Lai et al., 2021), and Japan (Hibiya et al., 2022).

Although the public health measures implemented to reduce COVID-19 transmission likely played a key role in these trends, changes in health care seeking behaviors, improved hand hygiene, and the redirecting of laboratory, health care, and public health resources to support the pandemic response are also potential contributing factors.

Clear trends in enteric disease surveillance have been documented in many countries, however, the impact of the COVID-19 pandemic on enteric diseases in Canada has yet to be formally assessed. This study is the first to describe the impact of the COVID-19 pandemic on enteric cases and clusters in Canada, for the period of March 2020 to December 2020, for *Escherichia coli* O157, non-O157 Shiga toxin–producing *Escherichia coli* (STEC), *Shigella*, *Salmonella*, and *Listeria monocytogenes*. In addition, this study aims to further explore the possible mechanisms for changes in reported enteric illness rates, including the impacts of changes to health care seeking behavior and restrictions on international travel.

## Materials and Methods

### Surveillance of enteric pathogens in Canada

The National Enteric Surveillance Program (NESP) collects and reports the number of key laboratory-confirmed enteric pathogens in Canada on a weekly basis. This program has been conducted since April 1997, through collaborations between the Public Health Agency of Canada (PHAC) and provincial public health laboratories. Timely surveillance and analysis occur for 14 enteric pathogens, including STEC, *Shigella*, *Salmonella*, and *L. monocytogenes*. Provincial public health laboratories across Canada send NESP weekly summaries of the number of enteric isolates from human patients that they have received for testing and/or confirmation.

Efforts are made to ensure that multiple isolates from a single case are removed, but it is possible there are duplicates in the data set. To improve readability, for the remainder of this article, the term “case” will be used instead of isolate. The data received are aggregated by province and pathogen. Further details on the enteric disease surveillance system that this study is based on, including clarifying which *E. coli* cases are included in the study, and how isolates are sequenced and placed into clusters, are included in Supplementary Data.

### Enteric cluster assessment

At the federal level, whole genome sequencing (WGS) data from PulseNet Canada are reviewed weekly by a team of epidemiologists within the PHAC to identify clusters that require further investigation to determine the vehicle of infection.

Clusters are classified as being (1) multijurisdictional, if there are human cases located in two or more provinces or territories or human cases in one province or territory and internationally or (2) single jurisdictional, if all human cases in the cluster are from one province or territory. Cases that do not group within 10 whole genome multi locus sequence typing (wgMLST) alleles of a multijurisdictional or single-jurisdictional cluster are considered sporadic cases. The wgMLST typing method was chosen for use as the results were shown to be highly congruent with the available epidemiological data during validation studies (Rumore et al., 2018).

Federal epidemiologists work closely with microbiologists and provincial/territorial epidemiologists when reviewing the data and identifying multijurisdictional clusters of interest. Exposure data may be collected from local/provincial health authorities on cases in specific clusters to help determine the vehicle of infection. For the purposes of this study, multijurisdictional clusters were classified as being associated either with international travel or with domestic. The criteria used to identify a cluster as being associated with international travel can be found in the Supplementary Data. Single-jurisdictional clusters were not included in this subanalysis as they are not routinely analyzed in Canada at the federal level.

### COVID-19 stringency index

In response to the COVID-19 pandemic, governments around the world implemented a variety of public health measures to mitigate harms. To explore the possible impact of these public health measures on the rates of enteric infections in Canada, the Oxford COVID-19 Government Response Tracker was used (Hale et al., 2021). This tool tracks government policies and interventions using a systematic and standardized method to develop a composite index, called the “Stringency Index.” The index ranges from 0 to 100, with a higher score indicating a more stringent response. Time series data of Canada's stringency index were plotted with the incidence of enteric infections reported nationally in 2020 to visually explore whether any trends were observed.

### Incidence rate ratio analysis

To identify differences in the incidence of reported enteric infections, incidence rate ratios (IRRs) were calculated comparing rates in 2020 with those of an appropriate reference period. These calculations were conducted for each pathogen overall, by cluster classification, and by cluster categorization. To analyze the impact of COVID-19 more accurately, the period analyzed for these calculations was restricted to epidemiological weeks 12–52. This period was chosen as public health measures in Canada began to be implemented in week 12 of 2020, as can be observed in the spike in the stringency index.

For overall case counts, the reference period was the equivalent weeks from a 5-year timespan from 2015 to 2019. For all cluster-based analyses, the reference period was the equivalent weeks from a 2-year timespan from 2018 to 2019 as prospective WGS was initiated in Canada beginning in 2017/2018. Details on the calculation for the person time at risk for the IRR are detailed in the Supplementary Data. The IRR was then calculated using Stata 16 (Stata Corporation, USA).

## Results

### Surveillance of enteric pathogens in Canada

**FIG. 1.**
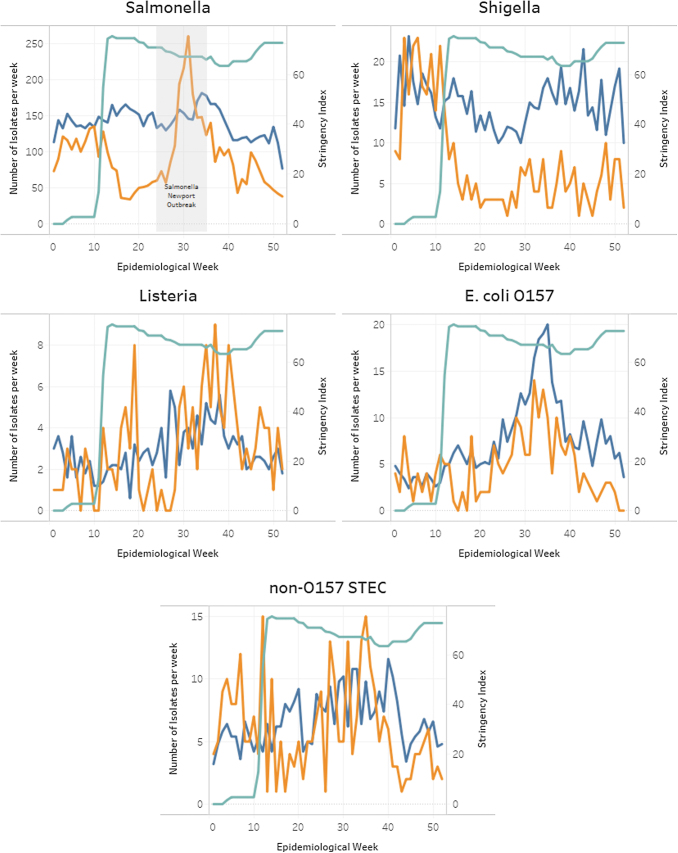
Comparison of weekly case counts for 2020 and the average weekly case counts for 2015–2019 for *Escherichia coli* O157, non-O157 STEC, *Shigella*, *Salmonella*, and *Listeria*, overlaid with the Canadian National Stringency Index for 2020. STEC, Shiga toxin**–**producing *Escherichia coli*.

Compared with the average number of cases reported by the provincial public health laboratories in the previous 5 years (9053), there was a 33% reduction in the total number of cases of enteric pathogens reported nationally in 2020 (6054). Results specific to each pathogen are shown in Figure 1. Reductions in the number of cases occurred, for most pathogens, after NESP week 12 that corresponded with a rapid increase in the COVID-19 stringency index, which indicates the implementation of numerous public health measures.

### Enteric cluster assessment

The estimated proportion of cases associated with single or multijurisdictional clusters compared with sporadic cases varied considerably based on pathogen (Table 1). *Escherichia coli* O157, *Shigella*, and *Salmonella* all had more than half the reported cases in 2020 associated with a single or multijurisdictional cluster, compared with non-O157 STEC and *Listeria* that both had ∼20% of cases associated with a cluster. Comparing the proportions of cases in clusters or sporadic cases between 2019 and 2020 showed little change. The most notable differences observed between 2019 and 2020 were the proportion of cases added to multijurisdictional clusters of non-O157 STEC, *Shigella*, and *L. monocytogenes.*

**Table 1.** Estimated Proportion of Reported Cases Identified as Part of a Single-Jurisdictional Cluster, Multijurisdictional Cluster, or as Sporadic, by Pathogen, for 2020 Compared with 2019 (95% Confidence Interval)

Pathogen	Salmonella		Shigella		Listeria monocytogenes		Escherichia coli O157		Non-O157 STEC	
Year	2019	2020	2019	2020	2019	2020	2019	2020	2019	2020
Multijurisdictional	0.535 (0.522–0.547)	0.559 (0.545–0.572)	0.449 (0.415–0.484)	0.529 (0.479–0.579)	0.207 (0.149–0.275)	0.114 (0.069–0.174)	0.363 (0.315–0.412)	0.346 (0.286–0.410)	0.15 (0.122–0.181)	0.081 (0.054–0.117)
Single jurisdictional	0.0387 (0.034–0.048)	0.0563 (0.0500–0.063)	0.144 (0.121–0.169)	0.109 (0.080–0.145)	0.063 (0.032–0.110)	0.076 (0.040–0.129)	0.234 (0.193–0.279)	0.236 (0.184–0.296)	0.139 (0.113–0.170)	0.113 (0.080–0.152)
Sporadic	0.427 (0.415–0.439)	0.385 (0.371–0.398)	0.407 (0.373–0.441)	0.361 (0.314–0.411)	0.73 (0.657–0.794)	0.81 (0.740–0.868)	0.403 (0.354–0.453)	0.418 (0.354–0.483)	0.711 (0.673–0.747)	0.806 (0.759–0.848)

STEC, Shiga toxin–producing *Escherichia coli.*

**FIG. 2.**
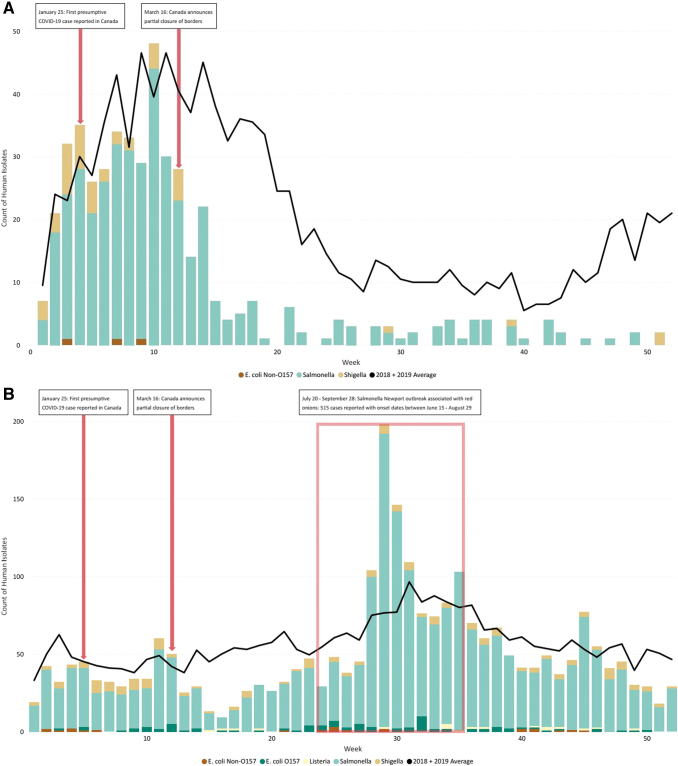
Number of human cases added to a multi-jurisdictional WGS cluster associated with international travel **(A)** and not associated with travel (domestic) **(B)** in 2020, compared to a two-year average (2018-2019).

In Canada, the number of reported cases associated with a multijurisdictional cluster linked to international travel typically follows a seasonal pattern with the highest number of cases in the winter months (January to March), however, travel cases are reported throughout the year. In 2020, there was a sharp decline in the number of reported cases associated with a travel cluster following the government directive to avoid all nonessential international travel (Fig. 2).

There was a similar decline in the number of reported cases associated with domestic clusters in March 2020 after the declaration of a national pandemic and the introduction of pandemic restrictions (Fig. 2), apart from a very large outbreak of *Salmonella* Newport associated with red onions in the summer of 2020 (Government of Canada, 2020c). Overall, the number of reported cases in a multijurisdictional cluster associated with international travel decreased 59.9% in 2020 compared with 2019 (455 cases reported in 2020 vs. 1134 cases in 2019) and the number of domestic cases decreased 10.3% (2574 cases reported in 2020 vs. 2869 cases in 2019).

### IRR analysis

**FIG. 3.**
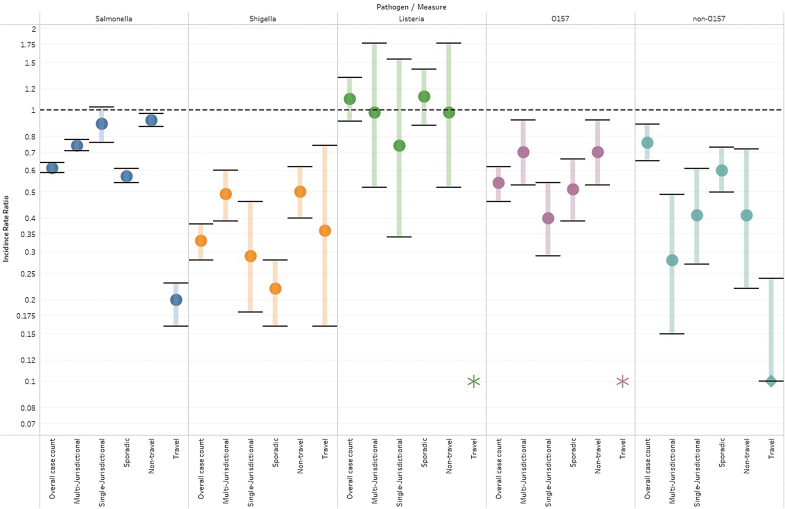
Incidence rate ratios comparing the rates between 2020 and referent periods for each pathogen.

There was a statistically significant reduction in the 2020 reported incidence rate compared with the respective reference period for 22 of the 28 IRRs calculated (*Escherichia coli* O157 and *Listeria* travel IRR is undefined). When examining trends in the IRRs across pathogens (Table 2; Fig. 3), IRRs for *Shigella* were found to be the lowest, with the IRR across all six subanalyses significant at the ≤0.05 alpha level. *Listeria* had the highest IRR, with none of the subanalyses finding a significant reduction, and the IRRs ranging from 0.74 to 1.12.

**Table 2.** Incidence Rate Ratios Comparing the Rates Between 2020 and Referent Periods for Each Pathogen

Pathogen	Pathogen									
	Salmonella		Shigella		Listeria monocytogenes		Escherichia coli O157		Non-O157 STEC	
	IRR (95% CI)	*p*	IRR (95% CI)	*p*	IRR (95% CI)	*p*	IRR (95% CI)	*p*	IRR (95% CI)	*p*
Overall										
Overall case count	0.61 (0.59–0.64)	<0.001	0.33 (0.28–0.38)	<0.001	1.10 (0.91–1.32)	0.313	0.54 (0.46–0.62)	<0.001	0.76 (0.65–0.89)	<0.001
Cluster type										
Multijurisdictional	0.74 (0.71–0.78)	<0.001	0.49 (0.39–0.60)	<0.001	0.98 (0.52–1.77)	0.961	0.70 (0.53–0.92)	0.009	0.28 (0.15–0.49)	<0.001
Single jurisdictional	0.89 (0.76–1.03)	0.104	0.29 (0.18–0.46)	<0.001	0.74 (0.34–1.54)	0.413	0.40 (0.29–0.54)	<0.001	0.41 (0.27–0.61)	<0.001
Sporadic	0.57 (0.54–0.61)	<0.001	0.22 (0.16–0.28)	<0.001	1.12 (0.88–1.42)	0.336	0.51 (0.39–0.66)	<0.001	0.60 (0.50–0.73)	<0.001
Cluster category										
Domestic	0.92 (0.87–0.97)	0.002	0.50 (0.40–0.62)	<0.001	0.98 (0.52–1.77)	0.961	0.70 (0.53–0.92)	0.009	0.41 (0.22–0.72)	<0.001
Travel	0.20 (0.16–0.23)	<0.001	0.36 (0.16–0.74)	0.002	NA		NA		0.00 (0.00–0.24)	<0.001

95% CI, 95% confidence interval; IRR, incidence rate ratio; NA, not available; STEC, Shiga toxin–producing *Escherichia coli*.

For each pathogen, when examining the IRRs across subanalyses, it is noteworthy that the IRRs for overall and the three cluster types (multijurisdictional, single jurisdictional, and sporadic) are generally similar in value (e.g., the IRRs for Salmonella are 0.61, 0.74, 0.89, and 0.57, respectively). This is not the case for cluster categorization, where for each pathogen the IRRs for domestic clusters and travel clusters are not similar (e.g., the IRRs for Salmonella are 0.92, and 0.20, respectively). For the three pathogens wherein both domestic and travel IRRs could be calculated, the travel clusters consistently had the lower IRR, suggesting there was a greater reduction in reported travel cases compared with domestic cases in 2020.

## Discussion

The total count of reported cases of enteric illness in Canada declined in 2020 compared with historical averages, and was the lowest total count in 23 years of monitoring enteric illness at the national level. Similar declines in reported enteric illness incidence in 2020 were observed in other countries including the United States that observed a 26% decrease in reported enteric illness incidence in 2020 (Ray et al., 2021). There are numerous hypotheses as to what caused these declines in the various jurisdictions, with many of them being related to the COVID-19 pandemic.

Examples include changes in health care seeking behavior (avoidance of health care settings) (Bassal et al., 2021; Love et al., 2022; Ray et al., 2021), changes to behaviors related to exposure (food consumption, indoor dining limits, event cancelations, and gathering restrictions) (Love et al., 2022; Steffen et al., 2020), and decreases in international travel (Love et al., 2022; Ray et al., 2021; Steffen et al., 2020). Our analyses examining variation in the components and pathogens may be able to provide some support for these hypotheses.

One of the hypothesized causes of the 2020 reduction in reported enteric illness is changes to health care seeking behavior, as there was a fear of being exposed to SARS-CoV-2 virus in health care settings (Roy et al., 2021). Of the pathogens included in this analysis, *Listeria* is the most likely to cause severe disease (De Valk et al., 2005). As health care seeking behavior is associated with disease severity (Thomas et al., 2013), the lack of reduction in *Listeria'*s 2020 reported incidence is possibly due to *Listeria* being likely to cause more severe illness compared with the other pathogens assessed.

It is possible that individuals with mild or moderate presentations of *Salmonella*, *Shigella*, *and E. coli* were less likely to seek care in 2020, and thus were not captured by surveillance, resulting in more notable decreases in their respective 2020 reported incidence rates. It is important to note that under this hypothesis, the drop in reported incidence rates does not necessarily suggest there was a drop in the true incidence rates.

Another possible rationale for the decline in enteric illness was behavior changes in the population. Untangling how the COVID-19 public health measures impacted behaviors related to the exposure to enteric pathogens is complex. The measures altered the eating habits of Canadians, including shifting diets and decreasing the amount of food consumed outside of the home as a result of limits on indoor dining and social events (Bender et al., 2022; Carroll et al., 2020). Whether these changes increased or decreased foodborne exposure to enteric pathogens would require further analysis and would likely be pathogen specific.

The impact of these measures on other routes of exposure, such as person-to-person exposure, can more easily be understood. The limitations of gatherings would reduce person-to-person contacts and thus exposure. This decline could provide the rationale as to why *Shigella* was found in our analysis to have greater reduction in reported incidence rates, compared with other pathogens. In Canada, a majority of *Shigella* cases (52.4%) are believed to be acquired through person-to-person transmission based on expert elicitation, whereas the other pathogens included in this study have substantially lower proportions of cases attributed to that transmission route (7.3–13.2%) (Butler et al., 2015).

An interesting finding from our analysis examining the IRRs of cluster classifications (single jurisdictional, multijurisdictional, and sporadic) was the lack of variation of these IRRs within pathogens. As previously mentioned, there are numerous ways in which the public health measures could have impacted exposure to enteric pathogens. The cluster classification analysis was thought to be one means of providing some evidence into these impacts.

For example, if the IRRs of the sporadic classification were found to be consistently lower than the other classifications, it could suggest that proportionally more cases were being acquired from isolated incidents such as improper food safety practices in a home, as opposed to larger outbreaks that could be associated with a food establishment. However, this was not our finding. There was relatively little variation in the IRRs between cluster classifications, suggesting that the public health measures impacted exposures that spanned the various cluster classifications.

The largest reduction observed across all analyses conducted was the decline in cases added to multijurisdictional clusters that were associated with international travel. This finding is not surprising given that the government of Canada implemented substantial restrictions on international travel and advised Canadians to avoid travel outside the country in March of 2020 (Government of Canada, 2020b). It is possible that the bulk of the reductions in observed incidence rates is attributable to reductions to international travel. FoodNet Canada is an enhanced sentinel-site surveillance system for enteric illnesses in Canada.

Comparing the proportional reductions observed in the reported national enteric incidence rates in 2020 with the proportion of cases reported to FoodNet Canada that were classified as being associated with international travel in 2019 showed similar percentages (*Salmonella*—39% compared with 33%; *Shigella*—67% compared with 52%; *Listeria*—10% compared with 0%; and *Escherichia coli* O157—46% and non-O157 STEC—24% compared with general STEC 35%) (Public Health Agency of Canada, 2020). Other jurisdictions also reported declines in the incidence rates of travel-associated enteric infections (Armistead et al., 2022; Ray et al., 2021).

It is important to highlight that some of the trends seen in this study may be impacted by non-COVID-19–related factors. For example, as a result of previous outbreaks of *Escherichia coli* O157 associated with romaine lettuce, import requirements were implemented for romaine lettuce originating from parts of California between October 7 and December 31, 2020, likely contributing to the reduced number of reported *Escherichia coli* O157 cases observed over the same time period (Government of Canada, 2020a). In contrast, a trend toward an increase in non-O157 STEC was noted before the onset of the pandemic, likely as a result of changes and advancements in laboratory testing (Chui et al., 2018).

### Limitations

There are some limitations to note while considering the study presented here. First, the number of cases reported nationally may be a subset of laboratory isolations and, therefore, may not be reflective of the true incidence of reported disease at the national level. Within NESP, *Salmonella* and *Escherichia coli* O157 cases are most representative of the true nationally reported incidence of disease compared with other pathogens. In contrast, although efforts are made to minimize over-reporting of organisms, this may occur due to the submission of multiple specimens from a single patient.

Second, efforts were made to use reference periods that were as robust as possible, however, due to Canada's transition to prospective WGS in 2017/2018, only a 2-year reference period was available for the cluster analysis. In comparison, the case-level analysis used a more robust 5-year reference period. Third, as already mentioned, other notable trends in enteric disease in Canada co-occurred with the COVID-19 pandemic and should be taken into consideration when interpreting the findings presented here. Lastly, a limitation of this study, and many like it, is the difficulty teasing apart the impacts of COVID-19 on enteric disease trends.

Although the stringency index was used as a proxy of all public health measures, we were not able to deduce the unique impacts of each public health measure, with the notable exception of international travel restrictions. We also were not able to deduce the unique impact of other COVID-19–related behavior changes, such as changes in health care seeking behaviors and hand washing. It is likely that the trends seen in this study are a result of a combination of COVID-19–related factors, and that the relative influence of each likely varied over time and geography.

## Conclusions

The findings of this study demonstrate that reported enteric illnesses in Canada were significantly impacted by the COVID-19 pandemic. Furthermore, analyzing the patterns observed in the reported incidence rates of enteric illnesses in 2020 provided evidence to support possible mechanisms that could have caused the changes to reported incidence rates, including changes to health care seeking behavior and international travel restrictions. This information can also be useful in understanding other pathogens that may not have enhanced surveillance data.

For example, if future research corroborates the findings of this study, and evidence shows that the majority of the decline in most pathogens was the result of the near full cessation of international travel, pathogens that have annual count data before and during the pandemic could possibly estimate the proportion of cases that were associated with international travel, by examining the decline in cases during the pandemic. Continued research into the impacts of the COVID-19 pandemic and associated public health measures on enteric illnesses may provide evidence to inform future policies and interventions aimed at reducing the burden of enteric illnesses in Canada.

## Supplementary Material

Supplemental data

## References

[B1] Armistead I, Tran A, White AE, et al. Trends in outpatient medical-care seeking for acute gastroenteritis during the COVID-19 pandemic, 2020. Foodborne Pathog Dis 2022;19:290–292. 35020464 10.1089/fpd.2021.0099

[B2] Bassal R, Keinan-Boker L, Cohen D. A significant decrease in the incidence of Shigellosis in Israel during COVID-19 pandemic. IJERPH 2021;18:3070. 33809746 10.3390/ijerph18063070 PMC8002282

[B3] Bender KE, Badiger A, Roe BE, et al. Consumer behavior during the COVID-19 pandemic: An analysis of food purchasing and management behaviors in US households through the lens of food system resilience. Socio-Econ Plan Sci 2022;82:101107. 10.1016/j.seps.2021.101107 PMC9192140 35721385

[B4] Butler AJ, Thomas MK, Pintar KD. Expert elicitation as a means to attribute 28 enteric pathogens to foodborne, waterborne, animal contact, and person-to-person transmission routes in Canada. Foodborne Pathog Dis 2015;12:335–344. 25835810 10.1089/fpd.2014.1856

[B5] Carroll N, Sadowski A, Laila A, et al. The impact of COVID-19 on health behavior, stress, financial and food security among middle to high income Canadian families with young children. Nutrients 2020;12:2352. 32784530 10.3390/nu12082352 PMC7468859

[B6] Chui L, Christianson S, Alexander D, et al. CPHLN recommendations for the laboratory detection of Shiga toxin-producing *Escherichia coli* (O157 and non-O157). CCDR 2018;44:304–307. 30996693 10.14745/ccdr.v44i11a06 PMC6449107

[B7] Cucinotta D, Vanelli M. WHO declares COVID-19 a pandemic. Acta Biomed 2020;91:157. 32191675 10.23750/abm.v91i1.9397 PMC7569573

[B8] de Miguel Buckley R, Trigo E, De La Calle-Prieto F, et al. Social distancing to combat COVID-19 led to a marked decrease in food-borne infections and sexually transmitted diseases in Spain. J Travel Med 2020;27:taaa134. 32841356 10.1093/jtm/taaa134 PMC7499626

[B9] De Valk H, Jacquet C, Goulet V, et al. Surveillance of Listeria infections in Europe. Eurosurveillance 2005;10:9–10. 10.2807/esm.10.10.00572-en 29208120

[B10] Detsky AS, Bogoch II. COVID-19 in Canada: Experience and response. JAMA 2020;324:743–744. 32790824 10.1001/jama.2020.14033

[B11] Government of Canada. New import requirement: romaine from parts of California must be tested for *E. coli*. Ottawa, Ontario. 2020a. Available from: https://www.canada.ca/en/food-inspection-agency/news/2020/09/new-import-requirement-romaine-from-parts-of-california-must-be-tested-for-e-coli.html [Last accessed: June 30, 2022].

[B12] Government of Canada. Prime Minister announces new actions under Canada's COVID-19 response. Ottawa, Ontario. 2020b. Available from: https://pm.gc.ca/en/news/news-releases/2020/03/16/prime-minister-announces-new-actions-under-canadas-covid-19-response [Last accessed: June 30, 2022].

[B13] Government of Canada. Public Health Notice: Outbreak of Salmonella infections linked to red onions imported from the United States. 2020c. Available from: https://www.canada.ca/en/public-health/services/public-health-notices/2020/outbreak-salmonella-infections-under-investigation.html [Last accessed: July 17, 2022].

[B14] Hale T, Angrist N, Goldszmidt R, et al. A global panel database of pandemic policies (Oxford COVID-19 Government Response Tracker). Nat Hum Behav 2021;5:529–538. 33686204 10.1038/s41562-021-01079-8

[B15] Hibiya K, Iwata H, Kinjo T, et al. Incidence of common infectious diseases in Japan during the COVID-19 pandemic. PLoS One 2022;17:e0261332. 35020724 10.1371/journal.pone.0261332 PMC8754328

[B16] Lai C-C, Chen S-Y, Yen M-Y, et al. The impact of the coronavirus disease 2019 epidemic on notifiable infectious diseases in Taiwan: A database analysis. Travel Med Infect Dis 2021;40:101997. 33640476 10.1016/j.tmaid.2021.101997 PMC7905388

[B17] Love NK, Elliot AJ, Chalmers RM, et al. Impact of the COVID-19 pandemic on gastrointestinal infection trends in England, February–July 2020. BMJ Open 2022;12:e050469. 10.1136/bmjopen-2021-050469 PMC8968111 35314468

[B18] Public Health Agency of Canada. FoodNet Canada tables and figures 2019. Government of Canada. Guelph, Ontario. 2020. Available from: https://publications.gc.ca/site/fra/9.892483/publication.html [Last accessed: June 30, 2022].

[B19] Ray LC, Collins JP, Griffin PM, et al. Decreased incidence of infections caused by pathogens transmitted commonly through food during the COVID-19 pandemic—Foodborne diseases active surveillance network, 10 US sites, 2017–2020. MMWR 2021;70:1332. 34555002 10.15585/mmwr.mm7038a4 PMC8459900

[B20] Roy CM, Bollman EB, Carson LM, et al. Assessing the indirect effects of COVID-19 on healthcare delivery, utilization and health outcomes: A scoping review. Eur J Public Health 2021;31:634–640. 33755130 10.1093/eurpub/ckab047 PMC8083627

[B21] Rumore J, Tschetter L, Kearney A, et al. Evaluation of whole-genome sequencing for outbreak detection of Verotoxigenic *Escherichia coli* O157: H7 from the Canadian perspective. BMC Genomics 2018;19:1–13. 30514209 10.1186/s12864-018-5243-3 PMC6278084

[B22] Steffen R, Lautenschlager S, Fehr J. Travel restrictions and lockdown during the COVID-19 pandemic—Impact on notified infectious diseases in Switzerland. J Travel Med 2020;27:taaa180. 33152761 10.1093/jtm/taaa180 PMC7543597

[B23] Thomas MK, Murray R, Flockhart L, et al. Estimates of the burden of foodborne illness in Canada for 30 specified pathogens and unspecified agents, circa 2006. Foodborne Pathog Dis 2013;10:639–648. 23659355 10.1089/fpd.2012.1389 PMC3696931

[B24] Wang L-P, Han J-Y, Zhou S-X, et al. The changing pattern of enteric pathogen infections in China during the COVID-19 pandemic: A nation-wide observational study. Lancet Reg Health Western Pac 2021;16:100268. 10.1016/j.lanwpc.2021.100268 PMC8450280 34568854

