# Supplementary material for: Impact of the COVID-19 Pandemic on the Reported Incidence of Select Bacterial Enteric Diseases in Canada, 2020
Source: Foodborne Pathog Dis. 2023 Mar 9;20(3):81–9. doi: 10.1089/fpd.2022.0064 (PMC9997028; doi:10.1089/fpd.2022.0064)
Supplement: Supplemental data [file Supp_DataS1.docx]

Supplemental Material

# Details on Enteric Disease Surveillance in Canada

## *E. coli* clarification

Unless otherwise noted, it is assumed that all samples of *E. coli* reported by the provincial laboratories are Shiga toxigenic *Escherichia coli* (STEC). Some provincial laboratories may identify *E. coli* cases using culture-independent diagnostic tests (CIDT). These tests are PCR-based and identify organisms without an isolate cultured. Non-typed STEC, CIDT positive for STX/STEC and non-STEC cases are not routinely reported by all the provinces and territories. Therefore, these *E. coli* cases were removed from our analysis.

## Isolate sequencing and cluster definitions

STEC*,* *Shigella*, *Salmonella,* and *Listeria monocytogenes* cases are sequenced at the National Microbiology Laboratory or by a PulseNet Canada certified provincial laboratory. The sequences are uploaded to PulseNet Canada and compared nationally using whole genome multi-locus sequencing typing (wgMLST) within a central BioNumerics v7.6.3 database (Applied Maths, USA). PulseNet Canada cluster codes are assigned to  *E. coli,* *Shigella*, *Salmonella,* and *Listeria* when 2 or more cases group together within 10 wgMLST allele differences within a specified time period (120 days for *Listeria*, 60 days for all other pathogens). Cluster codes are assigned to more common *Salmonella* serotypes including Typhimurium, Enteritidis and Heidelberg only when 3 or more cases group together within 10 wgMLST allele differences and 2 of the 3 cases are within 5 wgMLST alleles of each other. Once identified, clusters remain active indefinitely and allele ranges may be expanded or minimized based on laboratory, epidemiologic or food safety evidence.

# Criteria for a cluster being classified as being associated with international travel

A cluster was classified as associated with international travel if:

1. there was sufficient epidemiological evidence to suggest that travel outside of Canada was the source of the cluster (e.g., a substantial number of cases in the cluster reported travelling to the same country during their exposure period);
2. the cluster is within 10 wgMLST alleles of a known travel cluster; or
3. 3) the serotype is known to not be endemic to Canada (i.e., *Salmonella* Typhi or Paratyphi A).

Any cluster that did not meet one of these three criteria, including those with unknown exposures, was classified as domestic. Single-jurisdictional clusters were not included in this sub-analysis as they are not routinely analyzed in Canada at the federal level.

# Person-time at risk calculation for incidence rate

The person-time at risk for the incidence rate ratio calculation was obtained by taking the sum of the Canadian population on July 1st of each year, obtained from Statistics Canada, for the period of the rate (detailed in Table 1) and then multiplying by 41/52 to compensate for data representing only 41 of 52 weeks in a year.

Table 1. Calculating person-time at risk for incidence rate ratio calculation

| **Incidence Rate** | **Period** | **Total Canadian Population** | | **Compensation for partial year** | **Time at risk in person-years** |
| --- | --- | --- | --- | --- | --- |
|  |  | **Calculation** | **Total** |  |  |
| 2020 | 2020 | 38,037,204 | 38,037,204 | X 41/52 | 29,990,872 |
| Case count reference (overall analysis) | 2015-2019 | 35,702,908 +  36,109,487 +  36,545,236 +  37,065,084 +  37,601,230 | 183,023,945 | X 41/52 | 144,307,341 |
| Cluster reference (analysis by cluster classification and cluster categorization) | 2018-2019 | 37,065,084 +  37,601,230 | 74,666,314 | X 41/52 | 58,871,517 |
